# The Influence of Performance-Contingent Rewards on Proactive and Responsive Creativity: Dual-Path Mediating Effects of Work Motivation

**DOI:** 10.3389/fpsyg.2022.812298

**Published:** 2022-03-11

**Authors:** Chunling Li, Xinqing Jiang, Hui He, Xiying Zhang

**Affiliations:** Business School, Beijing Technology and Business University, Beijing, China

**Keywords:** performance-contingent rewards, autonomous motivation, controlled motivation, proactive creativity, responsive creativity, ambidexterity

## Abstract

Creativity has become prevalent in the routine work of knowledge employees in contemporary enterprises. From the perspective of ambidexterity, drawing upon expectancy theory and self-determination theory (SDT), the present study highlights the driver behind proactive and responsive creativity. Using two-stage longitudinal data collected from 373 knowledge employee-supervisor dyads in information and manufacturing companies in China, the results show that: (1) performance-contingent rewards have an inverted U-shaped influence on proactive creativity and a U-shaped influence on responsive creativity; (2) performance-contingent rewards have an inverted U-shaped influence on autonomous and controlled motivation; (3) autonomous motivation has a positive influence on proactive creativity, but controlled motivation has a negative influence on responsive creativity; (4) autonomous and controlled motivation play a partly mediating mechanism in the non-linear effects of performance-contingent rewards on proactive and responsive creativity, respectively. Managers should enhance the ambidextrous ability to deal with proactive and responsive creativity and establish an applied and dynamic policy of performance-contingent rewards intensity to drive ambidextrous creativity.

## Introduction

Nowadays, knowledge employees have comprised the majority of employees since knowledge-intensive jobs are prevalent in organizations (Drucker, 2001, p. 135–159; Oldham and Hackman, 2010). Creativity is one of the most critical determinants for knowledge employees’ productivity and the survival of enterprises in the future (Drucker, 2001, p. 135–159; Anderson et al., 2014). All employees are encouraged to participate in creativity fully by offering extrinsic rewards in many enterprises. Performance-contingent rewards and creativity-contingent rewards are two main kinds of extrinsic rewards. Performance-contingent rewards are defined as pay offered for **employees** routine performance indicators; by contrast, creativity-contingent rewards are pay offered only based on **employees** creative indicators (Byron and Khazanchi, 2012). Performance-contingent rewards have become the most popular incentive plan in organizations in the new economic era (Gerhart, 2017; Ma and Ma, 2018). Given continuous creativity being a part of routine work, the relationships between performance-contingent rewards and **employees** creativity may be strengthened more closely than ever. It is more worthy of discussing how performance-contingent rewards influence creativity (Gerhart and Fang, 2015).

Many scholars and practitioners have shown interest in whether extrinsic rewards enhance or hinder individual creative behavior in organizations for more than fifty years, and there has been considerable research (Byron and Khazanchi, 2012; Malik and Butt, 2017). Most studies have confirmed that creativity-contingent rewards can promote creativity (Byron and Khazanchi, 2012; Yoon et al., 2015). However, the results of empirical research on how performance-contingent rewards affect creativity have been still inconsistent. There are four different conclusions: performance-contingent rewards have a weakening effect on creativity (Byron and Khazanchi, 2012; Lee and Meyer-Doyle, 2017), performance-contingent rewards can promote creativity (Eisenberger and Aselage, 2009); performance-contingent rewards have an inverted U shape relationship with creativity (Zhang and Long, 2013a; Liu et al., 2014); performance-contingent rewards have no significant effect on creativity (Zhang et al., 2015a,b). These controversies lie in the following two important shortcomings in the existing studies.

Firstly, because of historically assuming creativity as a unitary construct (Unsworth, 2001; Gilson and Madjar, 2011), most of the literature emphasized on how the different types of extrinsic rewards (such as performance-contingent, creativity-contingence) impact creativity (Byron and Khazanchi, 2012; Malik and Butt, 2017). Nevertheless, only a few studies were concerned with the effect of similar rewards on the different types of creativity. Zhang and Long (2013b) and Zhang et al. (2014) examined how to pay for performance (PFP) triggers explorative and exploitative creativity, incremental and radical creativity. Malik and Butt (2017) claimed it is necessary to classify creativity to promote this research.

The study of Unsworth (2001) divided creativity into proactive and responsive creativity. Proactive and responsive creativity exhibits paradoxical goals and tasks of creativity (Gibson and Birkinshaw, 2004), namely ambidexterity of individual creativity. Enterprises have different needs for proactive and responsive creativity in various tasks and work contexts (Sung et al., 2017; Hwang and Choi, 2020). It is a field with great potential for creative research (Anderson et al., 2014). However, there is a lack of empirical research on how performance-contingent rewards trigger proactive and responsive creativity, so it cannot guide managerial practice.

Secondly, most existing studies focus on the direct impact of extrinsic rewards on creativity, but their mediating mechanism has been received little attention (Yoon et al., 2015; Amabile and Pratt, 2016; Malik and Butt, 2017). Among the mediators studied, the mediating role of intrinsic motivation was mainly concerned and highlighted whether the intrinsic motivation was crowded out or not (Eisenberger and Aselage, 2009; Zhang et al., 2015a). Specifically, the informational feedback of extrinsic rewards promoted intrinsic motivation, thus stimulating creativity; on the contrary, the controlling feedback of extrinsic rewards undermined intrinsic motivation, thus inhibiting creativity (Amabile, 1996, p. 116–122, 175; Malik and Butt, 2017). To our knowledge, the dichotomy of intrinsic motivation is difficult to explain the psychological states of performance-contingent rewards affecting proactive and responsive creativity.

We introduce ambidexterity, which provides a new research paradigm to fill the two gaps. On the one hand, by extending the creativity from a unidimensional construct to ambidextrous, we explore how performance-contingent rewards influence proactive and responsive creativity drawing on expectancy theory. On the other hand, based on self-determination theory (SDT), we focus on a dual-path mediating model of the effects of performance-contingent rewards on proactive and responsive creativity through autonomous and controlled motivation. To sum up, we investigate the psychological states of autonomous and controlled motivation induced by performance-contingent rewards, which predicts how individuals will take proactive or responsive behavior to ambidextrous creative needs. Our theoretical assumptions are empirically validated using data of 373 employee-supervisor dyads from manufacturing and information companies in China.

## Theoretical Background and Hypotheses

### Perspective of Ambidexterity

Ambidexterity has become an important research paradigm in management studies, focusing on conflicting activities in an organizational dynamic. In general, ambidexterity is an evolving concept (Turner et al., 2013; Papachroni et al., 2015). Initially, ambidexterity is an organization’s capability to simultaneously balance conflicting and different activities (e.g., exploration and exploitation) in a trade-off context (Rothaermel and Alexandre, 2009; Turner et al., 2013). Later, Papachroni et al. (2015) introduced the perspective of paradox and pointed out that ambidexterity refers to elements that seem contradictory yet closely interrelated and usually appear simultaneously in an organization. According to Papachroni et al. (2015), ambidexterity of psychological cognitions, behaviors, or activities are no longer two opposing poles of an either-or relationship but the coexisting relationship (Bledow et al., 2009; Papachroni et al., 2015).

This study adopts the view of Papachroni et al. (2015) instead of the dichotomy. From the perspective of ambidexterity, this study develops an integrative framework that accounts for how performance-contingent rewards induce simultaneously different forms of work motivation and employees’ creativity.

### Proactive Creativity and Responsive Creativity

From the perspective of the ambidextrous classification of creativity, the existing research has broadly focused on exploratory and exploitative creativity or radical and incremental creativity (Jansen et al., 2006; Gilson and Madjar, 2011; Turner et al., 2013; Anderson et al., 2014; Papachroni et al., 2015). Proactive and responsive creativity have gained relatively less research attention. Unsworth (2001) first divided creativity into proactive and responsive creativity from the two dimensions of open-closed problem-type and internal-external driver-type. Proactive creativity refers to individuals initiatively and voluntarily addressing creative ideas or solutions to open problems such as those to be discovered without specific requirements. Conversely, responsive creativity refers to individuals submitting creative ideas or solutions in response to closed problems such as existing specific problems or external requirements. Sung et al. (2017) pointed out proactive and responsive creativity is the most differentiable two-dimensional concept with the least overlap. They adopted the definition of Unsworth (2001) and developed a unidimensional measurement scale of proactive and responsive creativity. The discriminant validity and convergent validity of the scale were tested and verified by Hwang and Choi (2020).

In recent years, Chinese scholars categorized creativity into two forms based on the type of autonomous-controlled, namely, proactive and reactive innovation behavior (Zhao et al., 2014, 2015; Yang et al., 2019). Although Chinese scholars did not consider the difference of problem types, the motivation-type of Chinese scholars and driver-type of Unsworth (2001) are both based on SDT’s work motivation. Whereas, in our opinion, empirical research is needed to support the relationship between two types of work motivation and ambidextrous creativity.

### Performance-Contingent Rewards and Proactive-Responsive Creativity

Performance-contingent rewards refer to pay offered for employees’ routine performance indicators (Byron and Khazanchi, 2012), a variable portion of employees’ regular total monetary compensation (Milkovich and Wigdor, 1991, p. 9–11; Gerhart, 2017). The proportion of performance-contingent rewards in the total monetary compensation is performance-contingent rewards intensity (Gerhart, 2017), an objective measurement variable of performance-contingent rewards in this study. In general, performance-contingent rewards can also be regarded as PFP because most plans of both are the same. However, PFP is a broader term to denote any compensation plan relating to performance (Gerhart, 2017). For example, creativity-contingent rewards belong to PFP but cannot be included in performance-contingent rewards (Byron and Khazanchi, 2012). Given the rigor and complexity of the evaluation procedure, creativity-contingent rewards are not generally considered as a part of the regular total compensation package in Chinese enterprises.

Expectancy theory (Vroom, 1964, p. 79–86) identifies three key components that result in individuals choosing one certain behavioral option, (1) valence is the value the individuals place on specific rewards, namely, outcome, (2) expectancy is the belief that greater effort will result in better performance, (3) instrumentality is the belief that the individuals will receive rewards if the performance is met. When deciding a behavioral choice, individuals try to select valence, expectancy, and instrumentality with the greatest amount of incentives. Performance-contingent rewards have an incentive effect on employees because they believe that performance-contingent rewards can establish a more direct and clear connection amongst effort, performance, and reward to encourage employees to show the expected behavior (Gerhart and Rynes, 2003).

Creativity is an important factor affecting employees’ work performance (Drucker, 2001, p. 135–159). Drawing on expectancy theory, extrinsic rewards positively affect creativity only when employees value the given rewards and strongly believe in attaining the creative goals (Malik et al., 2015). Moreover, proactive and responsive creativity are risky and require employees to make more efforts, so employees expect to get more rewards.

Research showed that the receipt of reward for high performance increased their performance pressure which, in turn, could promote creativity (Eisenberger and Aselage, 2009). Increasing performance-contingent rewards intensity will likely change employees’ behavior (Gerhart et al., 2009). With performance-contingent rewards intensity gradually ascending, performance pressure becomes gradually increases which, in turn, was likely positively related to proactive and responsive creativity. However, the connection between work performance and proactive creativity is vague for open tasks without specific requirements. Moreover, with the increase of performance-contingent rewards intensity, the vagueness of the relationship would cause employees to become less confident in obtaining performance-contingent rewards by proactive creativity and less effort in proactive creativity. It can be thus inferred that with the increase of the intensity of performance-contingent rewards, proactive creativity first rises at a decreasing rate, after reaching a maximum, declines at an increasing rate. Namely, this study predicts that an inverted U-shaped relationship may exist between performance-contingent rewards and proactive creativity.

In contrast, the connection between work performance and responsive creativity is clear for close tasks with responsible requirements. Moreover, with the increase of performance-contingent rewards intensity, clearness of the relation would cause employees to become more confident in obtaining performance-contingent rewards by responsive creativity and more effort in responsive creativity. It can be thus inferred that with the increase of the intensity of performance-contingent rewards, employees always tend to show more responsive creativity. The previous results showed that PFP has an inverted U-shaped relationship with explorative creativity and positively affects exploitative creativity (Zhang and Long, 2013b). Therefore, we hypothesize:

*H1a: performance-contingent rewards have an inverted U-shaped relationship with proactive creativity.*

*H1b: performance-contingent rewards have a significant positive influence on responsive creativity.*

### Dual Mediating Effects of Autonomous-Controlled Motivation

#### Motivation Within Self-Determination Theory

SDT initially discerns three significant types of motivation (Ryan and Deci, 2000; Ryan and Deci, 2017, p. 14–16). Intrinsic motivation, namely intrinsic regulation, refers to individuals doing an activity for their intrinsic interest and enjoyment. Extrinsic motivation is defined as engaging in an activity to attain instrumental outcomes, such as gaining external rewards and approval, avoiding criticism or punishments, or achieving a valued goal. Amotivation, namely non-regulation is defined as individuals lacking the intention to engage in an activity.

SDT further specifies varied subtypes of extrinsic motivation, which differ in their internalization (Deci and Ryan, 2000; Ryan and Deci, 2017, p. 14–16). Internalization is the internal psychological process in which individuals take in values, beliefs, or regulatory structures from external factors and transform them into one’s own. The degree of internalization reflects four regulating processes: external regulation, introjected regulation, identified regulation, and integrated regulation (Deci and Ryan, 2000; Gagné and Deci, 2005; Ryan and Deci, 2017, p. 179–215). External regulation, which is entirely non-internalized, refers to the individual behavior dependent on or motivated by external instrumental factors such as reward or punishment. Introjected regulation, a partial and incomplete internalization, refers to the individual behavior in assimilating external rules or values for internally pressuring forces, such as boosting one’s self-esteem and avoiding guilt and anxiety. Identified regulation, a form of volitional internalization, refers to individuals genuinely identifying with the value or meaning of behavior for their own. Integrated regulation, which is the fullest type of internalization, means that the individual fully internalizes the extrinsic motivation and identifies with the value of the activity. However, integrated regulation is still regarded as extrinsic motivation because the motivation is characterized by the individual goals of being instrumentally crucial in the activity, not by the individual being interested (Ryan and Connell, 1989; Gagné and Deci, 2005; Ryan and Deci, 2017, p. 179–215). Therefore, these different forms of regulations, ranging from non-regulation, external to internal regulation, fall along a continuum of relative autonomy, reflecting individual behavior change from nonself-determined to self-determined (Deci and Ryan, 2000; Ryan and Deci, 2017, p. 179–215).

In view of the above, SDT has shifted the distinction of motivation from intrinsic vs. extrinsic to autonomous vs. controlled motivation based on the concept of internalization and types of regulation dimension (Ryan and Deci, 2017, p. 14–16). Autonomous motivation is an individual doing an activity with a full sense of volition and endorsement, consisting of intrinsic, identified, and integrated regulation. In contrast, controlled motivation is an individual engaging in an activity to feel pressured or forced to do so, including introjected and external regulations (Vansteenkiste et al., 2004; Deci and Ryan, 2008).

SDT describes the different forms of extrinsic motivation and the process of external factors (such as extrinsic rewards) that promote or hinder internalization and integration. The autonomous-controlled continuum is the core concept to distinguish types of motivation, developing out of intrinsic and extrinsic motivation (Vansteenkiste et al., 2004; Ryan and Deci, 2017, p. 14–16). SDT is a popularly cited motivation theory in many domains, such as work, education (Howard et al., 2017). Compared with other theories, the types of motivation with SDT can better explain the relationship between extrinsic rewards and creativity (Malik and Butt, 2017). This study thus uses autonomous-controlled motivation as the mediating variable of performance-contingent rewards affecting proactive and responsive creativity.

#### Performance-Contingent Rewards and Autonomous-Controlled Motivation

A substantial literature has suggested motivation is like a relatively stable trait, but also it is a temporary state influenced significantly by the immediate situation; work environments with strong motivational structures can change an individual motivational trait (Amabile, 1993). That is to say, motivational states are subject to change, depending on changes in the immediately environmental factors (Amabile and Pratt, 2016). The effects of contextual factors (i.e., performance-contingent rewards) on human behavior are determined by individual differences that cause different cognitions and attributions of the same context (Ajzen, 1991). From the perspective of ambidexterity and SDT, the autonomous and controlled work motivation is in a state of change with the different intensities of performance-contingent rewards.

Performance-contingent rewards, as an external instrumental factor, have three distinct kinds of functional significance depending on the individual’s interpretation: informational, controlling, and amotivating (Deci and Ryan, 1985a, p. 60–122; Ryan and Deci, 2017, p. 159–160). The meaning of the informational aspect is that individuals feel positive or effectance-relevant feedback in the context of choice. The meaning of the controlling aspect is that individuals feel negative or pressured feedback in the context of no choice. The meaning of the amotivating aspect is that individuals feel unable to attain effectance. That functional significance impacts satisfying basic psychological needs differently, which are necessary for any type of motivation (Deci and Ryan, 2000, 2008; Gagné et al., 2015).

Under the lower performance-contingent rewards intensity, with intensity gradually increasing, the informational feedback of performance-contingent rewards to employees gradually strengthens. Employees will get more recognition by meeting performance indicators, which can more strongly enhance employees’ feeling of competence and self-determination; put in this, more employee’s psychological need is satisfied, more autonomous motivation is facilitated. Studies have confirmed that external factors such as performance-contingent rewards, autonomous support, and perceived organizational support satisfy basic psychological needs and have a positive effect on employees’ autonomous motivation (Eisenberger and Aselage, 2009; Chambel et al., 2015; Güntert, 2015; Nie et al., 2015; Van Schie et al., 2015). On the other hand, as the intensity rises, uncertainty and risk of performance-contingent rewards also increase. The controlling feedback of performance-contingent rewards will continue to advance, which satisfaction of basic psychological needs will continue to diminish, so the controlled motivation may also continue to increase (Eisenberger and Aselage, 2009; Gubler et al., 2016; Deci et al., 2017). Moreover, Amabile (1993) presented that “high levels of intrinsic and high levels of extrinsic motivation can be made to coexist through some situational factors temporarily.”

When performance-contingent rewards intensity continues to increase and enters a higher level, the uncertainty and risk of performance-contingent rewards continue ascending, resulting in excessive-performance pressure. The informational feedback of performance-contingent rewards turns to decrease gradually; as a result, the satisfaction of basic psychological needs begins to decline gradually, and autonomous motivation will turn to be gradually undermined (Deci et al., 1999). At the same time, the controlling feedback of performance-contingent rewards continues strengthening until performance pressure exceeds the tolerance of employees. When individuals perceive the failure of continuous negative performance feedback or fail to achieve the desired goals, the satisfaction of basic psychological needs can thus be more seriously thwarted. That is, individual being a sense of powerlessness and incompetence, frustration and self-depreciation, poorer motivation or amotivation occurs (Deci and Ryan, 1985a, p. 60–122; Ryan and Deci, 2017, p. 159–160). Consequently, with performance-contingent rewards intensity gradually increasing, the controlled motivation will ultimately turn to be a declining trend (Deci and Ryan, 1985b). Therefore, we hypothesize:

*H2a: performance-contingent rewards have an inverted U-shaped relationship with autonomous motivation.*

*H2b: performance-contingent rewards have an inverted U-shaped relationship with controlled motivation.*

#### Autonomous-Controlled Motivation and Proactive-Responsive Creativity

According to SDT, the intrinsic regulation, integrated regulation, and identified regulation of autonomous motivation enable employees to show self-identity and voluntary behavior (Ryan and Connell, 1989; Deci and Ryan, 2000; Ryan and Deci, 2000; Gagné and Deci, 2005; Deci and Ryan, 2008; Ryan and Deci, 2017, p. 179–215). The employees driven by autonomous motivation are more likely to accept challenges and pursue a sense of achievement. Thus, they will actively explore potential problems or opportunities of their jobs and put forward creative solutions, exhibiting more proactive creativity. Although there is a lack of research on the influence of autonomous motivation on proactive creativity, dozens of empirical studies generally supported that autonomous motivation has a significant positive effect on employees’ positive behavior or attitude (Deci and Ryan, 2008; Deci et al., 2017; Ryan and Deci, 2017; Zhang, 2019). For example, autonomous motivation positively predicts proactive behavior, employee vitality, affective commitment (Gagné et al., 2015; Zhang and Wu, 2016), work effort (Gagné et al., 2015; Kuvaas et al., 2016), goal progress (Koestner et al., 2008), work engagement (Lopes and Chambel, 2017), and altruism, civic virtue, job satisfaction (Battistelli et al., 2013). Autonomous motivation thus has a significant positive influence on proactive creativity.

In contrast, both the external regulation and the introjected regulation of controlled motivation enable employees to avoid anxiety and guilt or perform for some purpose (Ryan and Connell, 1989; Deci and Ryan, 2000, 2008; Ryan and Deci, 2000, 2017, p. 179–215; Gagné and Deci, 2005). The employees driven by controlled motivation are more likely to submit creative ideas or solutions to specific problems or external demands, exhibiting responsive creativity. Similarly, there is currently a lack of research on the influence of controlled motivation on responsive creativity. However, limited studies have shown that controlled motivation may significantly reinforce negative behaviors or attitudes. For example, controlled motivation positively predicts emotional exhaustion and turnover intention (Gagné et al., 2015; Kuvaas et al., 2016), often stimulates the minimum required effort (Ryan and Deci, 2017). Responsive creativity should not be regarded as an absolutely negative behavior, but it is a negative behavior to a certain degree compared with proactive creativity.

In addition, some studies showed that controlled motivation has a significant positive influence on positive behavior or attitude, such as proactive behavior (Gagné et al., 2015; Zhang and Wu, 2016), work effort (Kuvaas et al., 2016), which can be explained by internalization. From the perspective of ambidexterity and SDT, well-internalized activities are regulated by autonomous motivation. The conclusion that “controlled motivation has a significant positive influence on positive behavior or attitude” should be somewhat attributed to autonomous motivation than controlled motivation. So it can be said that responsive creativity should be driven by controlled motivation. We hypothesize:

*H3a: Autonomous motivation has a significant positive influence on proactive creativity.*

*H3b: Controlled motivation has a significant positive influence on responsive creativity.*

#### Mediating Effects of Autonomous-Controlled Motivation

In sum, according to SDT, when informational feedback of performance-contingent rewards supports the fulfillment of employees’ basic psychological needs, autonomous motivation can be enhanced, thus leading to drive proactive creativity. In contrast, when controlled feedback of performance-contingent rewards thwarts the fulfillment of employees’ basic psychological needs, controlled motivation can be enhanced, thus driving responsive creativity. No empirical study has shown that autonomous-controlled motivation is mediating between performance-contingent rewards and proactive-responsive creativity. Nevertheless, studies have demonstrated strong relations between autonomous-controlled motivation and various other antecedent and outcome variables over the past 30 years (Howard et al., 2017). Some studies showed that autonomous motivation mediates the relationship between performance-contingent rewards and work effort (Kuvaas et al., 2016), behavioral control, promotion focus and creativity (Li et al., 2016; Ren et al., 2017), organizational commitment and civic virtue, job satisfaction (Battistelli et al., 2013). The other studies showed that controlled motivation mediates the relationship between performance-contingent rewards and turnover intention (Kuvaas et al., 2016), prevention focus, and creative behavior (Li et al., 2016). These findings provide indirect literature support for the mediating role of autonomous-controlled motivation between performance-contingent rewards and proactive-responsive creativity. Thus, we hypothesize:

*H4a: Autonomous motivation mediates the relationship between performance-contingent rewards and proactive creativity.*

*H4b: Controlled motivation mediates the relationship between performance-contingent rewards and responsive creativity.*

**FIGURE 1:**
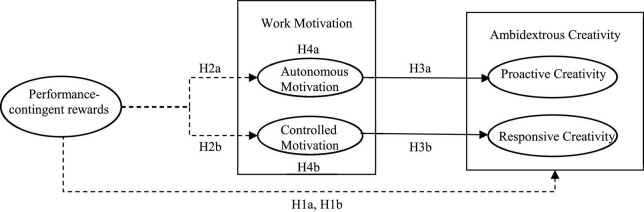
Theoretical framework model (full line means linear relations while imaginary line means non-linear relations).

The theoretical framework model is depicted in Figure 1.

## Materials and Methods

### Sample and Data Collection

We sampled knowledge employees from manufacturing and information companies such as computer, communications, and electronic equipment manufacturing, software development, Internet search in eastern and central China. Based on 163 valid questionnaires of pre-survey, we found the reliability of all variable measurements had been acceptable. From January to February 2020, we conducted two formal surveys with an interval of 2 weeks. The data were collected from subordinates and their supervisors to reduce common method bias. In Time-1, subordinates answered their demographics and assessed performance-contingent rewards intensity, autonomous and controlled motivation. Two weeks later, in Time-2, supervisors evaluated their subordinates’ proactive and responsive creativity. A total of 373 matched subordinate-supervisor data were returned for final analysis.

Among the samples, 52% were men, 48% were women; 13.7% were from 18 to 25 years of age, 57.9% were from 26 to 35 years of age, 22.8% were from 36 to 45 years of age, and 5.6% were aged over 45. All participants were well educated and qualified to be knowledge employees; 2 years of college accounted for 7.2%, bachelor’s for 64.3%, master’s for 26%, doctor’s for 2.4%. As for company tenure, 3.8% less than 1 year, 35.1% from 1 to 3 years, 44.8% from 4 to 9 years, 12.3% from 10 to 15 years, 3.5% from 16 to 20 years, and 0.5% more than 20 years. The job type of technical and product R&D accounted for 56.6%, operations/sales and supporting for 43.4%. We stressed anonymity and confidentiality in every questionnaire and informed consent form for each participant and stated that all data in the survey was only for research purposes.

### Variable Measurement

All measurement scales are drawn from previous studies. All measures were translated to Chinese by following a procedure of standard translation-back-translation to ensure the congruence of English versions of the scales. In order to control common method bias, we adopted the pre-control measures such as the random assignment of items and hidden meanings of items. Items of autonomous-controlled motivation scale and proactive-responsive creativity scale were rated on Likert six-point scale ranging from “strongly disagree” (1) to “strongly agree” (6).

Performance-contingent rewards intensity, as the objective measurement variable of performance-contingent rewards, was measured by a single item from Du (2009). Subordinates were asked to answer a question that “What is the percentage of your performance-contingent rewards in your salary on average monthly?” (a) 0–5%; (b) 6–15%; (c) 16–30%; (d) 31–50%; (e) 0.51–69%; (f) 70–84%; (g) 85–94%; (h) 95–100%.” The above eight interval values were not evenly divided to measure the extreme intensity values better. The median value of each interval was taken as the intensity value in the analysis.

The autonomous-controlled motivation was measured with a 16-item scale proposed by Gagné et al. (2015). The autonomous motivation scale included six items of identified regulation and intrinsic regulation, such as “Because I have fun doing my job,” and the Cronbach’s α was 0.91. The Controlled motivation scale included ten items of introjected and external regulation, such as “Because I have to prove to myself that I can,” and the Cronbach’s α was 0.85.

Proactive-responsive creativity was measured by a 10-item scale from Sung et al. (2017) rated by the supervisors. The proactive creativity scale included five items, such as “This employee suggests useful ideas and solutions even without a specific problem to solve,” and the Cronbach’s α was 0.928. Responsive creativity included five items, such as “This employee exerts acceptable creative efforts but rarely exceeds requirements,” and the Cronbach’s α was 0.896.

Control variables included gender, age, education, company tenure, and job type in consistence with previous studies (Zhang and Long, 2013a,b; Liu et al., 2014; Zhang et al., 2015a,b; Sung et al., 2017), to reduce the possibility that demographic variables which may impact creativity would confuse the relations examined in the present study.

## Results

### Confirmatory Factor Analysis

A confirmatory factor analysis was conducted to test the discriminant validity of the four latent variables (autonomous motivation, controlled motivation, proactive creativity, and responsive creativity) in [AMOS 23.0, International Business Machines Corporation (IBM), Armonk, NY, United States] Three alternative models were constructed based on the four-factor model (all variables were separated independently). Table 1 exhibits the test results. Fit indexes showed an adequate fit for our hypothesized four-factor model, with the data (χ^2^/df = 2.3 < 3, RMSEA = 0.059 < 0.08, RMR = 0.052 < 0.08, NFI = 0.892 > 0.8, CFI = 0.936 > 0.9, GFI = 0.87 > 0.8, AGFI = 0.844 > 0.8). Four-factor model’s fitting degree is better than other alternative models, indicating that the study variables have good discriminant validity.

**TABLE 1:** The result of confirmatory factor analysis of the models.

Factor structure	χ^2^	df	χ^2^/df	NFI	CFI	GFI	AGFI	RMSEA	RMR
Four-factor model	673.956	293	2.300	0.892	0.936	0.870	0.844	0.059	0.052
Three-factor model	857.288	296	2.896	0.863	0.905	0.827	0.794	0.071	0.066
Two-factor model	1108.189	298	3.719	0.822	0.863	0.772	0.732	0.085	0.078
One-factor model	2597.616	299	8.688	0.584	0.611	0.437	0.339	0.144	0.150

*N = 373; three-factor model (proactive creativity + responsive creativity, autonomous motivation, controlled motivation); two-factor model (proactive creativity + responsive creativity, autonomous motivation + controlled motivation); single-factor model merges all variables into one factor.*

### Descriptive Statistics and Correlations

Table 2 exhibits descriptive statistics and bivariate correlations of all the variables. According to descriptive statistical analysis, the mean of performance-contingent rewards is 0.27, and the mean of autonomous motivation, controlled motivation, proactive creativity, and responsive creativity is 4.71, 4.65, 4.49, and 2.75, respectively. An inspection of the correlations shows that performance-contingent rewards were not significantly related to proactive creativity, responsive creativity, autonomous motivation and controlled motivation (*r* = 0.097, *r* = −0.066, *r* = 0.021, *r* = 0.059, *p* > 0.05). It is proved that there was no linear relationship between performance-contingent rewards and the four variables, respectively, but other types of relationships might exist or not. Additionally, autonomous motivation was significantly related to proactive creativity (*r* = 0.554, *p* < 0.01), and controlled motivation was significantly related to responsive creativity (*r* = −0.225, *p* < 0.01).

**TABLE 2:** Descriptive analysis and correlations among variables.

Variables	Mean	*SD*	G	A	E	T	J	PCR	AM	CM	PIB
Gender	1.48	0.500	1								
Age	2.20	0.741	–0.040	1							
Education	2.24	0.612	–0.099	–0.035	1						
Tenure	2.78	0.876	−0.117*	0.515**	0.026	1					
Job type	0.57	0.496	−0.187**	–0.007	0.126*	0.079	1				
PCR	0.27	0.183	–0.082	0.096	0.101	0.149**	0.006	1			
AM	4.71	0.837	–0.050	0.069	–0.030	0.081	0.084	0.021	1		
CM	4.65	0.634	–0.039	0.037	0.040	0.088	0.153**	0.059	0.647**	1	
PC	4.49	0.986	–0.020	0.067	−0.121*	0.102*	0.179**	0.097	0.554**	0.381**	1
RC	2.75	0.932	–0.037	–0.049	0.178**	–0.088	−0.110*	–0.066	−0.371**	−0.225**	-0.795**

N = 373; PCR, performance-contingent rewards; AM, autonomous motivation; CM, controlled motivation; PC, proactive creativity; RC, responsive creativity. Significance level: *P < 0.05, **p < 0.01.

### Hypothesis Tests

#### Analytic Strategy

Hierarchical regression analysis is adopted to test the mediation of hypotheses using [SPSS 21.0, International Business Machines Corporation (IBM), Armonk, NY, United States]. Referring to Baron and Kenny (1986) methods of testing mediating mechanisms, the following conditions should hold: firstly, the independent variable significantly affects the dependent variable; then, the independent variable significantly affects the mediator; finally, the mediator significantly affects the dependent variable. Furthermore, when the independent and mediator are added in the regression equation to explain the dependent variable in the meantime, the effect of the mediator is significant if the independent variable has no effect (all mediating effects) or less effect (partial mediating effects). All of the testing results are shown in Tables 3, 4.

**TABLE 3:** Hierarchical regression analysis for the mediating effect of autonomous motivation.

Variable model	Proactive creativity					Autonomous motivation		
	M1	M2	M3	M4	M5	M6	M7	M8
Gender	0.012	0.019	0.042	0.029	0.048	–0.031	–0.031	–0.011
Age	0.022	0.019	0.000	0.001	–0.012	0.040	0.040	0.024
Education	−0.145**	−0.155**	−0.170**	−0.123**	−0.141**	–0.043	–0.044	–0.056
Tenure	0.080	0.068	0.070	0.053	0.043	0.052	0.050	0.053
Job type	0.194***	0.196***	0.193***	0.151**	0.153***	0.080	0.080	0.077
PCR		0.101	0.662***		0.408**		0.011	0.492**
PCR^2^			−0.588***		−0.327*			−0.504**
AM				0.535***	0.517***			
*R* ^2^	0.062	0.072	0.106	0.343	0.362	0.016	0.017	0.042
△*R*^2^	0.049	0.056	0.089	0.332	0.348	0.003	0.000	0.024
*F*	4.825***	4.698***	6.206***	31.879***	25.872***	1.230	1.029	2.297*

N = 373; PCR, performance-contingent rewards; AM, autonomous motivation. Significance level: *P < 0.05, **p < 0.01, ***p < 0.001.

**TABLE 4:** Hierarchical regression analysis for the mediating effect of controlled motivation.

Variable model	Responsive creativity					Controlled motivation		
	M9	M10	M11	M12	M13	M14	M15	M16
Gender	–0.054	–0.059	–0.077	–0.055	–0.074	–0.001	0.001	0.017
Age	0.000	0.003	0.017	0.000	0.014	0.000	–0.002	–0.014
Education	0.192***	0.200***	0.211***	0.197***	0.212***	0.020	0.015	0.006
Tenure	–0.088	–0.079	–0.081	–0.072	–0.067	0.076	0.070	0.072
Job type	−0.137**	−0.140**	−0.137**	−0.107*	−0.109*	0.144**	0.145**	0.143**
PCR		–0.078	−0.509**		−0.429**		0.047	0.414*
PCR^2^			0.451**		0.377*			−0.385*
CM				−0.212***	−0.194***			
R2	0.059	0.065	0.086	0.103	0.121	0.030	0.032	0.047
△R2	0.046	0.050	0.068	0.088	0.102	0.016	0.016	0.028
F	4.616***	4.247***	4.884***	6.983***	6.284***	2.237	1.995	2.550*

N = 373; PCR, performance-contingent rewards; CM, controlled motivation. Significance level: *P < 0.05, **p < 0.01, ***p < 0.001.

In addition, according to the curve mediation test procedure provided by Hayes and Preacher (2010), we used the MEDCURVE SPSS macro to conduct Bootstrap sampling 5,000 times on the whole sample. The instantaneous indirect effects of performance-contingent rewards on proactive-responsive creativity produced by autonomous-controlled motivation are estimated in the case of three representative values of performance-contingent rewards (mean and mean ± 1 *SD*), as shown in Table 5.

**TABLE 5:** The instantaneous indirect effect of performance-contingent rewards on proactive-responsive creativity through autonomous-controlled motivation at different values of performance-contingent rewards.

Motivation	PCR	Instantaneous indirect effect	95%CI
AM	0.0920 (Mean − 1 *SD*)	1.0561	[0.2430, 2.0663]
	0.2747 (Mean)	0.4281	[0.0176, 0.9123]
	0.4573 (Mean + 1 *SD*)	−0.2000	[−0.5953, 0.1459]

CM	0.0920 (Mean −1 *SD*)	−0.3234	[−0.7437, −0.0693]
	0.2747(Mean)	−0.1537	[−0.3667, −0.0218]
	0.4573 (Mean + 1 *SD*)	0.0160	[−0.0931, 0.1507]

*N = 373; PCR, performance-contingent rewards; AM, autonomous motivation; CM, controlled motivation; Coefficients are unstandardized; 95% CI, 95% confidence intervals with lower and upper limits; Bootstrap samples = 5,000.*

#### Main Effects

Table 3 exhibits the results of hierarchical regression analysis on the main effects of performance-contingent rewards on proactive creativity. Models 2 and 3 examine H1a. Model 2 shows that performance-contingent rewards in the linear regression equation have no significant effect on proactive creativity (β = 0.101, *p* > 0.05). Moreover, inputting the quadratic term of performance-contingent rewards in Model 3, performance-contingent rewards in the linear equation have a significant positive association with proactive creativity (β = 0.662, *p* < 0.001), and the quadratic term of performance-contingent rewards have a significant negative association with proactive creativity (β = −0.588, *p* < 0.001). Combining the results of Models 2 and 3 produces that performance-contingent rewards have an inverted U-shaped relationship with proactive creativity, then H1a is confirmed.

Table 4 exhibits the results of hierarchical regression analysis on the main effects of performance-contingent rewards on responsive creativity. Models 10 and 11 examine H1b. Model 10 shows that performance-contingent rewards in the linear regression equation have no significant effect on responsive creativity (β = −0.078, *p* > 0.05). Furthermore, inputting the quadratic term of performance-contingent rewards in Model 11, performance-contingent rewards in the linear regression equation have a significant negative association with responsive creativity (β = −0.509, *p* < 0.01), and the quadratic term of performance-contingent rewards have a significant positive association with responsive creativity (β = 0.451, *p* < 0.01). Combining the results of Models 10 and 11 produces that performance-contingent rewards have a U-shaped relationship with responsive creativity, then H1b is rejected.

#### Mediating Effects

Mediating effects of autonomous motivation. Firstly, Model 7 and Model 8 examine H2a. Model 7 shows that performance-contingent rewards in the linear regression equation have no significant effect on autonomous motivation (β = 0.011, *p* > 0.05). Moreover, inputting the quadratic term of performance-contingent rewards in Model 8, performance-contingent rewards in the linear equation have a significant positive association with autonomous motivation (β = 0.492, *p* < 0.01), and the quadratic term of performance-contingent rewards have a significant negative association with autonomous motivation (β = −0.504, *p* < 0.01). Combining the results of Models 7 and 8 produces that performance-contingent rewards have an inverted U-shaped relationship with autonomous motivation, then H2a is confirmed.

Secondly, Model 4 examines H3a. Model 4 shows that autonomous motivation has a significant positive influence on proactive creativity (β = 0.535, *p* < 0.001), H3a is confirmed.

Thirdly, Models 3 and 5 examine H4a. Inserting autonomic motivation into Model 5, the association between the quadratic term of performance-contingent rewards and proactive creativity is still significant (β = −0.327, *p* < 0.05), but the absolute value of the coefficient has gone down (from 0.588 to 0.327). Thereby, autonomous motivation partially mediates the relationship between performance-contingent rewards and proactive creativity, H4a is confirmed. Then, the MEDCURVE macro was used to conduct Bootstrap sampling 5,000 times on the whole sample, as shown in Table 5. When the value of performance-contingent rewards is Mean −1SD or Mean, the instantaneous indirect effect is positive and significant (indirect effect = 1.0561, 95% CI [0.2430, 2.0663]; indirect effect = 0.4281, 95% CI [0.0176, 0.9123]). However, when the value of performance-contingent rewards is Mean + 1 *SD*, the instantaneous indirect effect is not significant (indirect effect = −0.2, 95% CI [−0.5953, 0.1459]). This result indicates that performance-contingent rewards affect proactive creativity through autonomous motivation at a low and moderate level of performance-contingent rewards intensity but not at a high level. Taken together, these results further support H4a.

Mediating effects of controlled motivation. Firstly, Models 15 and 16 examine H2b. Model 15 shows that performance-contingent rewards in the linear regression equation have no significant effect on controlled motivation (β = 0.047, *p* > 0.05). Furthermore, inputting the quadratic term of performance-contingent rewards in Model 16, performance-contingent rewards in the linear regression equation have a significant positive association with controlled motivation (β = 0.414, *p* < 0.05), and the quadratic term of performance-contingent rewards have a significant negative association with controlled motivation (β = −0.385, *p* < 0.05). Combining the results of Models 15 and 16 produces that performance-contingent rewards have an inverted U-shaped relationship with controlled motivation, H2b is confirmed.

Secondly, Model 12 examines H3b. Model 12 shows that controlled motivation has a significant negative influence on responsive creativity (β = −0.212, *p* < 0.001), H3b is rejected.

Thirdly, Models 11 and 13 examine H4b. Inserting controlled motivation into Model 13, the association between the quadratic term of performance-contingent rewards and responsive creativity is still significant (β = 0.377, *p* < 0.05), but the absolute value of the coefficient has gone down (from 0.451 to 0.377). Thereby, controlled motivation partially mediates the relationship between performance-contingent rewards and responsive creativity, H4b is confirmed. Then, the MEDCURVE macro was used to conduct Bootstrap sampling 5,000 times on the whole sample, as shown in Table 5. When the value of performance-contingent rewards is Mean − 1 *SD* or Mean, the instantaneous indirect effect is negative and significant (indirect effect = −0.3234, 95% CI [−0.7437, −0.0693]; indirect effect = −0.1537, 95% CI [−0. 3667, −0.0218]). However, when the value of performance-contingent rewards is Mean + 1 *SD*, the instantaneous indirect effect is not significant (indirect effect = 0.016, 95% CI [−0.0931, 0.1507]). This result indicates that performance-contingent rewards affect responsive creativity through controlled motivation at a low and moderate level of performance-contingent rewards intensity but not at a high level. Taken together, these results further support H4b.

### *Post hoc* Analysis

We conduct two sets of *post hoc* analyses to check the robustness of our results.

Firstly, we test the mediating role of autonomous motivation in the relationship between performance-contingent rewards and responsive creativity. On the one hand, autonomous motivation has a significant negative influence on responsive creativity (β = −0.356, *p* < 0.001). On the other hand, a new model is formed by adding autonomous motivation to Model 11. The new model shows that the association between the quadratic term of performance-contingent rewards and responsive creativity is not significant (β = 0.280, *p* > 0.05), but the relationship between performance-contingent rewards in the linear regression equation and responsive creativity is still significant (β = −0.342,*p* < 0.05). Then, the MEDCURVE macro was used to conduct Bootstrap sampling 5,000 times on the whole sample, as shown in Table 6. When the value of performance-contingent rewards is Mean − 1 *SD* or Mean, the instantaneous indirect effect is negative and significant (indirect effect = −0.6569, 95% CI [−1.3549, −0.1499]; indirect effect = −0.2663, 95% CI [−0.5942, −0.009]). However, when the value of performance-contingent rewards is Mean + 1 *SD*, the instantaneous indirect effect is not significant (indirect effect = 0.1244, 95% CI [−0.0913, 0.3958]). This result indicates that performance-contingent rewards affect responsive creativity through autonomous motivation at a low and moderate level of performance-contingent rewards intensity but not at a high level. Thereby, autonomous motivation partially mediates the relationship between performance-contingent rewards and responsive creativity. Nevertheless, the existing theories and literature are most difficult to make convincing inferences about the negative impact of autonomous motivation on responsive creativity. Shadish et al. (2002) argued that empirical studies of causality between two variables must be based on key theoretical mechanisms to clarify how the causality arises. The mediation of autonomous motivation between performance-contingent rewards and responsive creativity thus would likely be inferred not to be accepted theoretically.

**TABLE 6:** The instantaneous indirect effect of performance-contingent rewards on responsive-proactive creativity through autonomous-controlled motivation at different values of performance-contingent rewards.

Motivation	PCR	Instantaneous indirect effect	95%CI
AM	0.0920 (Mean −1 *SD*)	−0.6569	[−1.3549, −0.1499]
	0.2747 (Mean)	−0.2663	[−0.5942, −0.0090]
	0.4573 (Mean + 1 *SD*)	0.1244	[−0.0913, 0.3958]

CM	0.0920 (Mean −1 *SD*)	0.5985	[0.1310, 1.1913]
	0.2747 (Mean)	0.2845	[0.0138, 0.5976]
	0.4573 (Mean + 1 *SD*)	−0.0296	[−0.2496, 0.1851]

*N = 373; PCR, performance-contingent rewards; AM, autonomous motivation; CM, controlled motivation; Coefficients are unstandardized; 95% CI, 95% confidence intervals with lower and upper limits; Bootstrap samples = 5,000.*

Secondly, we test the mediating role of controlled motivation in the relationship between performance-contingent rewards and proactive creativity. On the one hand, controlled motivation has a significant positive influence on proactive creativity (β = 0.361, *p* < 0.001). On the other hand, a new model is formed by adding controlled motivation to Model 3. The new model shows that the association between the quadratic term of performance-contingent rewards and proactive creativity is still significant (β = −0.457, *p* < 0.01), but the absolute value of the coefficient has gone down (from 0.588 to 0.457). Then, the MEDCURVE macro was used to conduct Bootstrap sampling 5,000 times on the whole sample, as shown in Table 6. When the value of performance-contingent rewards is Mean − 1 *SD* or Mean, the instantaneous indirect effect is significant (indirect effect = 0.5985, 95% CI [0.131, 1.1913]; indirect effect = 0.2845, 95% CI [0.0138, 0.5976]). However, when the value of performance-contingent rewards is Mean + 1 *SD*, the instantaneous indirect effect is not significant (indirect effect = −0.0296, 95% CI [−0.2496, 0.1851]). This result indicates that performance-contingent rewards affect proactive creativity through controlled motivation at a low and moderate level of performance-contingent rewards intensity but not at a high level. Thereby, controlled motivation partially mediates the relationship between performance-contingent rewards and proactive creativity. Nevertheless, based on the internalization of SDT (Deci and Ryan, 2000; Ryan and Deci, 2017, p. 183–184), controlled motivation positive influence on proactive creativity would be actually regarded as autonomous motivation’s effect on proactive creativity when controlled motivation is internalized (Gagné et al., 2015; Kuvaas et al., 2016; Zhang and Wu, 2016). Therefore, the mediation of controlled motivation between performance-contingent rewards and proactive creativity would still be insufficient in theory and literature. According to Shadish et al. (2002), this path thus would likely be inferred not to be accepted theoretically.

## Conclusion and Discussion

### Conclusion

The empirical results show that H1a, H2a, H2b, H3a, H4a, and H4b have been confirmed except H1b and H3b. The results support the research model of dual-path mediation in the non-linear effects of performance-contingent rewards on proactive and responsive creativity, just as the following: (1) performance-contingent rewards have an inverted U-shaped influence on proactive creativity and a U-shaped influence on responsive creativity; (2) performance-contingent rewards have an inverted U-shaped influence on autonomous motivation, autonomous motivation positively influences proactive creativity, and autonomous motivation partly mediates the relationship between performance-contingent rewards and proactive creativity; (3) performance-contingent rewards have an inverted U-shaped influence on controlled motivation, controlled motivation negatively influences responsive creativity, and controlled motivation partly mediates the relationship between performance-contingent rewards and responsive creativity. In conclusion, performance-contingent rewards at an intermediate level of intensity would promote more proactive creativity. In contrast, performance-contingent rewards at a low and high level of intensity would drive more responsive creativity.

### Theoretical Contributions

Firstly, this study demonstrates that performance-contingent rewards can elicit ambidextrous creativity, advancing research on the relationship between extrinsic rewards and creativity. Previous research on proactive and responsive creativity classification showed much promise (Unsworth, 2001; Anderson et al., 2014; Sung et al., 2017; Hwang and Choi, 2020). Nevertheless, there have not been tests of proactive and responsive creativity in the relationship between extrinsic rewards and creativity (Malik and Butt, 2017). From the perspective of ambidexterity, we regard creativity as the organization’s demand for individual ambidextrous creativity instead of a unidimensional construct (Bledow et al., 2009). This result supports the conclusion of Zhang and Long (2013b) to some extent. Performance-contingent rewards have an inverted U-shaped influence on proactive creativity (H1a), supporting expectancy theory (Vroom, 1964). However, performance-contingent rewards have a U-shaped influence on responsive creativity; H1b is rejected. The two findings will be further explained appropriately in the following partly indirect effects.

Secondly, this study reveals a dual-path mediation of autonomous and controlled motivation in the non-linear relations, which adds new literature on the mediation between extrinsic rewards and creativity. According to the perspective of ambidexterity and SDT (Deci and Ryan, 1985a; Papachroni et al., 2015; Ryan and Deci, 2017), this study overcomes the dichotomous opposition of intrinsic motivation in the previous research (Yoon et al., 2015; Amabile and Pratt, 2016; Malik and Butt, 2017). On the one hand, the findings suggest an inverted U-shaped relationship between performance-contingent rewards and autonomous-controlled motivation (H2a, H2b), which indicate that autonomous and controlled motivation is rather not two oppositional poles of an either-or relationship, but a coexisting relation (Bledow et al., 2009; Papachroni et al., 2015).

On the other hand, this study indicates the psychological states of performance-contingent rewards affecting proactive-responsive creativity, which reasonably interprets the different non-linear relationship between performance-contingent rewards and proactive-responsive creativity. (1) Autonomous motivation partly mediates the relationship between performance-contingent rewards and proactive creativity (H4a). At the low and moderate levels of performance-contingent rewards intensity, autonomous motivation has a mediating effect. Meanwhile, as intensity increases, autonomous motivation shows an upward trend which, in turn, has a positive impact on proactive creativity (H3a), thus strengthening the positive impact of performance-contingent rewards on proactive creativity. Whereas at the high level of intensity, autonomous motivation has no mediating effect, performance-contingent rewards impacting proactive creativity is only a directly negative effect. Therefore, the total effect of performance-contingent rewards on proactive creativity is an inverted U-shaped relationship. (2) Controlled motivation partly mediates the relationship between performance-contingent rewards and responsive creativity (H4b). At the low and moderate levels of performance-contingent rewards intensity, controlled motivation has a mediating effect. Meanwhile, as intensity increases, controlled motivation shows an upward trend which, in turn, has a negative impact on responsive creativity (H3b is rejected), thus changing the positive impact of performance-contingent rewards on responsive creativity into the negative impact. Whereas at the high level of intensity, controlled motivation has no mediating effect, performance-contingent rewards impacting responsive creativity is only a directly positive effect. Therefore, the total effect of performance-contingent rewards on responsive creativity is a U-shaped relationship.

Furthermore, this study indicates that autonomous motivation positively influences proactive creativity while controlled motivation negatively influences responsive creativity. H3a is confirmed, which is consistent with the views of existing studies (Deci and Ryan, 2008; Deci et al., 2017; Ryan and Deci, 2017; Zhang, 2019). Although H3b is rejected, this finding supports Amabile’s view of motivation (1993) and the internalization of STD (Deci and Ryan, 1985a; Ryan and Deci, 2017). From this point, the higher the level of intrinsic motivation is, the more likely internalization is to occur. As autonomic motivation is enhanced, controlled motivation is more likely to be transformed into autonomic motivation, thus promoting proactive creativity and weakening responsive creativity. It explains why controlled motivation negatively affects responsive creativity. In this way, controlled motivation can trigger more responsive creativity only when controlled motivation is lower and more difficultly transformed into autonomous motivation.

### Implications for Practice

Firstly, managers can recognize the driving mechanism of proactive and responsive creativity from the perspective of ambidexterity. Proactive and responsive creativity reflects enterprises’ different needs for knowledge employee creativity in different situations (Unsworth, 2001; Sung et al., 2017; Hwang and Choi, 2020). With increasing uncertainty in the business environment and continuous creativity being a part of routine work, ambidextrous creativity frequently exists simultaneously and dynamically (Papachroni et al., 2015). It is critical for managers to deeply understand how performance-contingent rewards stimulate the partial indirect effect of autonomous-controlled motivation and engender different types of creativity. It can help the managers distinguish the two creativity and improve their ambidextrous ability to make and implement the policy of performance-contingent rewards.

Secondly, we suggest redesigning the applied and dynamic performance-contingent rewards intensity to guide the expected creativity of enterprises. If the informational and controlling aspect is interpreted as two ends of the performance-contingent rewards scale, the intensity is the fulcrum of the scale. It is vital to set appropriate incentive intensity for managers to balance the informational and controlling aspect to maximize proactive and responsive creativity. Thus, managers should redesign the policy of intensity according to the problem-type of the job tasks. As for the positions with non-routine and unstructured tasks, managers should set an intermediate level of intensity to encourage more proactive creativity. As for the positions with routine and structured tasks, managers should set a low or high level of intensity to trigger more responsive creativity. When job tasks change, managers should adjust the intensity of performance-contingent rewards to lead employees to transform between proactive and responsive creativity.

Additionally, organizations generally implement two or more kinds of performance-contingent rewards at present (Gerhart, 2017). The interview surveys of this study also report that Chinese enterprises carry out several performance-contingent rewards plans, such as R&D project bonus, commission, profit-sharing plan, and gainsharing plan. Employees typically combine rewards from multiple plans as part of their total compensation. Thereby, enterprises should set an intensity based on the total proportion of various forms of performance-contingent reward in the compensation package to keep it in the expected proportion.

### Limitations and Future Research Directions

First, there may be two interfering paths beyond those hypothesized mediating effects of work motivation. One may be the mediation of autonomous motivation in the impact of performance-contingent rewards on responsive creativity; the other may be controlled motivation in the impact of performance-contingent rewards on proactive creativity. Although the *post hoc* analyses show that this study would not be likely to accept the two paths theoretically, the validity of the conclusion in theory and empirical tests should be valuable to discuss in future research. In addition, this study also inferred the influence of excessive controlling aspect of performance-contingent rewards on amotivation, but did not propose a hypothesis and empirically test with limited by the research topic. Thus, future research will pay attention to the mediating effect of amotivation between performance-contingent rewards and creativity.

Second, this study ignored differences among subgroups of work motivation with variable-centered approaches. Variable-centered approaches assume that the population samples are homogenous, but there is more or less heterogeneity in reality. Therefore, person-centered approaches should be introduced with assuming the samples as heterogeneous in the future study. A latent profile analysis should be adopted to identify the different types of latent profiles of autonomous and controlled motivation (Wang and Hanges, 2011) and find the mediating mechanisms on performance-contingent rewards driving proactive and responsive creativity among different employee subgroups in further research.

Third, the sample data of this study were collected from knowledge employees in manufacturing and information enterprises in China. The relationship between work motivation and employees’ behavior in different countries or industries is different (Gagné et al., 2015). For example, Sung et al. (2017) found that compared with Korean employees, the positive effects of job complexity on psychological empowerment and cognitive overload among Swedish were slightly weaker but likely to exhibit more responsive creativity. Therefore, the psychological state of employees in various countries or industries to performance-contingent rewards may be different. It will be further verified whether the results of this study apply to other countries or industries. Future research should pay more attention to comparative studies of work motivation mediating mechanism effect on performance-contingent rewards affecting proactive and responsive creativity among samples from different countries or industries.

Fourth, this study did not concern different plans of performance-contingent rewards. Future research can extend to how different plans of performance-contingent rewards (such as R&D project bonus, commission, profit-sharing plan, and gainsharing plan) drive proactive and responsive creativity, which should be a potential direction.

## Data Availability Statement

The raw data supporting the conclusions of this article will be made available by the authors, without undue reservation.

## Author Contributions

CL: conceptualization, methodology, overall writing of the manuscript, resources, and funding acquisition. XJ: software, drafting of the early version, review, and editing. HH: supervision and funding acquisition. XZ: data curation and drafting of the early version. All authors contributed to the article and approved the submitted version.

## Conflict of Interest

The authors declare that the research was conducted in the absence of any commercial or financial relationships that could be construed as a potential conflict of interest.

## Publisher’s Note

All claims expressed in this article are solely those of the authors and do not necessarily represent those of their affiliated organizations, or those of the publisher, the editors and the reviewers. Any product that may be evaluated in this article, or claim that may be made by its manufacturer, is not guaranteed or endorsed by the publisher.
